# The nanoscaled metal-organic framework ICR-2 as a carrier of porphyrins for photodynamic therapy

**DOI:** 10.3762/bjnano.9.275

**Published:** 2018-11-30

**Authors:** Jan Hynek, Sebastian Jurík, Martina Koncošová, Jaroslav Zelenka, Ivana Křížová, Tomáš Ruml, Kaplan Kirakci, Ivo Jakubec, František Kovanda, Kamil Lang, Jan Demel

**Affiliations:** 1 Department of Materials Chemistry, Institute of Inorganic Chemistry of the Czech Academy of Sciences, Husinec-Řež 1001, 250 68 Řež, Czech Republic,; 2 Department of Solid State Chemistry, University of Chemistry and Technology, Technická 5, 166 28 Prague, Czech Republic,; 3 Department of Biochemistry and Microbiology, University of Chemistry and Technology, Technická 5, 166 28 Prague, Czech Republic,; 4 Department of Biotechnology, University of Chemistry and Technology, Technická 5, 166 28 Prague, Czech Republic

**Keywords:** metal-organic framework, phosphinic acid based MOF, photodynamic therapy, porphyrin, singlet oxygen

## Abstract

Nanosized porphyrin-containing metal-organic frameworks (MOFs) attract considerable attention as solid-state photosensitizers for biological applications. In this study, we have for the first time synthesised and characterised phosphinate-based MOF nanoparticles, nanoICR-2 (Inorganic Chemistry Rez). We demonstrate that nanoICR-2 can be decorated with anionic 5,10,15,20-tetrakis(4-R-phosphinatophenyl)porphyrins (R = methyl, isopropyl, phenyl) by utilizing unsaturated metal sites on the nanoparticle surface. The use of these porphyrins allows for superior loading of the nanoparticles when compared with commonly used 5,10,15,20-tetrakis(4-carboxyphenyl)porphyrin. The nanoICR-2/porphyrin composites retain part of the free porphyrins photophysical properties, while the photodynamic efficacy is strongly affected by the R substituent at the porphyrin phosphinate groups. Thus, phosphinatophenylporphyrin with phenyl substituents has the strongest photodynamic efficacy due to the most efficient cellular uptake.

## Introduction

Metal-organic frameworks (MOFs) are a class of crystalline coordination polymers possessing potential voids. Their structures combine inorganic nodes, metal centres forming so-called secondary building units (SBU), with organic linkers. The diversity of possible SBUs coupled with organic linkers of variable geometry enables the preparation of a large number of structures with tuneable pore sizes, topologies, and chemical nature [1–2]. Among them, MOFs with photoactivatable properties such as luminescence and photosensitization of singlet oxygen, O_2_(^1^∆_g_), are particularly attractive [3–5]. Singlet oxygen is a short-lived, highly oxidative species with bactericidal and virucidal properties [6]. The cytotoxic effect can be intentionally employed in anticancer treatment in the form of photodynamic therapy (PDT) [7–8].

The most commonly utilised photosensitizers in PDT are porphyrins or related compounds since they offer high quantum yields of O_2_(^1^∆_g_), chemical and photochemical stability, and absorb light between 600 and 900 nm, the region in which tissue transmits light best [9]. However, porphyrins tend to form aggregates in which the photosensitizing properties are lost [10]. In order to avoid porphyrin aggregation various supramolecular structures have been designed [11–13]. In this context, porphyrin-based MOFs offer unique systems in which a regular arrangement prevents porphyrins from aggregation whereas the porosity enables fast diffusion of the ground state O_2_(^3^Σ_g_) to and the excited O_2_(^1^∆_g_) from the solid photosensitizer [14–16].

In our recent work we have shown that microcrystalline porphyrin containing MOFs are poor O_2_(^1^∆_g_) photosensitizers [17], due to the combined effect of the quenching of excited states in tightly stacked porphyrin units and strong light absorption at the surface of microcrystalline particles, which results in a small portion of the molecules actively taking place in the photosensitizing process. One of the successful strategies to overcome these effects is the use of MOFs as nanoparticles, which also provides easier internalisation by cells [18]. Moreover, the downsizing of MOFs also facilitates the diffusion of O_2_(^1^∆_g_), and makes interactions with bulky biomolecules inside the cells more effective.

This concept was successfully applied using the UiO-66 family of MOFs: nanoparticles made of Hf_6_-based SBUs with dicarboxylic porphyrin or chlorin linkers [19–20], or using Zr_6_-based SBUs with 5,10,15,20-tetrakis(4-carboxyphenyl)porphyrin (TCPP), named PCN-224 and PCN-222 [21–22]. Zeng et al. extended the π system of TCPP by employing tetracarboxyphenyl benzoporphyrin, which increased the absorption in the red region of visible light [23]. Alternatively, the antitumor activity of porphyrinic PCN-224 was increased by combining photodynamic and photothermal effects with chemotherapy; in this case the MOF was deposited onto gold nanorods and impregnated with a chemotherapeutic agent [24]. Strong phototoxic effects were reported in all of these studies. However, the drawback of zirconium-based MOFs is the degradation in the presence of phosphate buffer (Figure S1, Supporting Information File 1) and therefore the mode of action is highly disputable [15].

**Figure 1:**
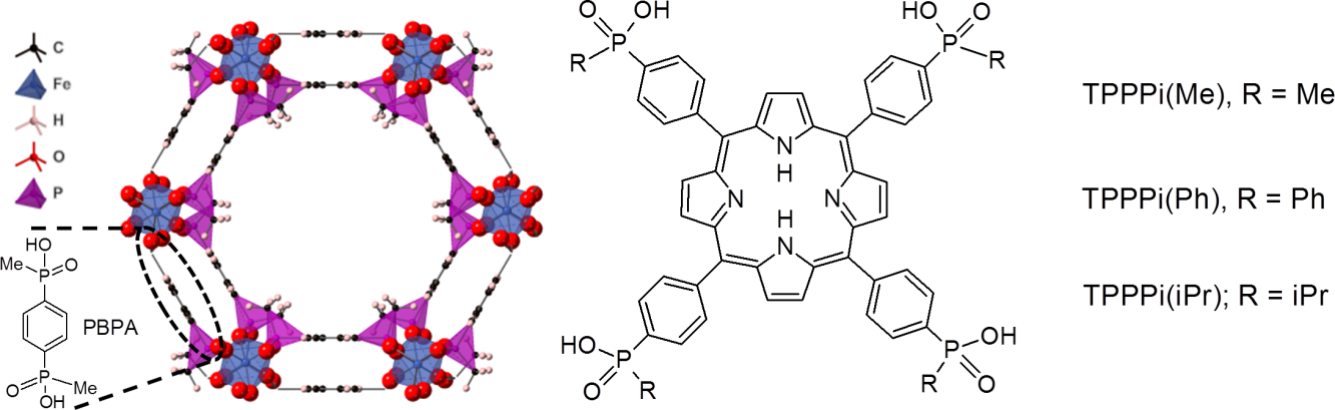
Structure of ICR-2 viewed along the *c* axis (left) and the structure of the modifying porphyrins (right). Colour coding: octahedrally coordinated iron atoms are blue and phosphinate tetrahedra are magenta, O (red), C (black), and H (white).

In this work, we employed metal-organic framework ICR-2 (ICR stands for Inorganic Chemistry Rez) constructed from Fe^3+^ and phenylene-1,4-bis(methylphosphinic acid) (PBPA) linkers [25]. ICR-2 in the microcrystalline form is stable in aqueous solutions even at high temperatures and partly retains its structure and porosity even after treatment with phosphate buffer saline (PBS) (Figure S1, Supporting Information File 1). We prepared ICR-2 nanoparticles (nanoICR-2) the surface of which we modified with three different anionic porphyrins forming stable colloids in absolute EtOH or *N*,*N*-dimethylformamide (DMF) (Figure 1). Importantly, the porphyrins on the nanoparticle surfaces retain their photophysical properties including O_2_(^1^∆_g_) generation. We demonstrate the photodynamic activity of these nanoICR-2/porphyrin composites on HeLa cells.

## Results and Discussion

### Preparation and characterisation

**Figure 2:**
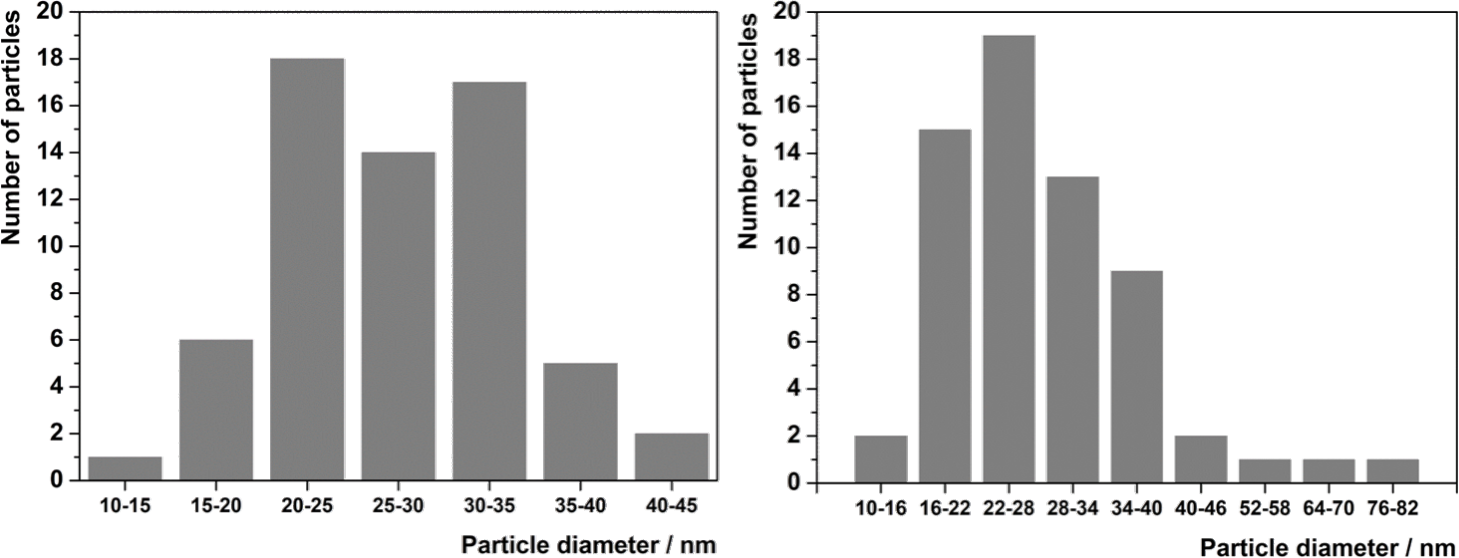
Particle size distributions of nanoICR-2 (left) and nanoICR-2/TPPPi(Ph) (right).

**Figure 3:**
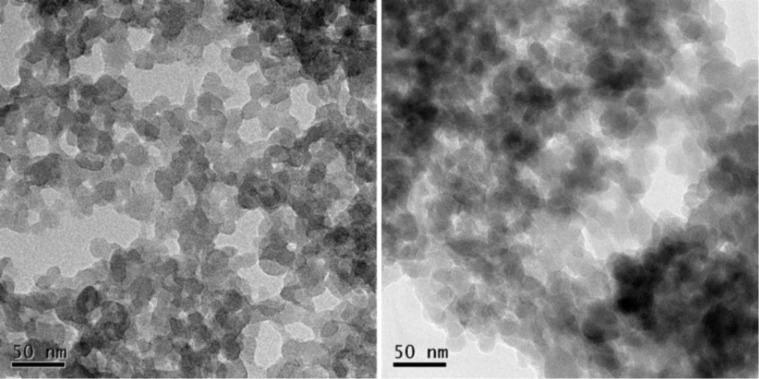
TEM images of parent nanoICR-2 (left) and nanoICR-2/TPPPi(Ph) (right). The scale bars represent 50 nm.

Various organic solvents and temperatures were screened for the successful preparation of nanoICR-2. The use of pure formamide (FA) or mixtures with DMF of more than 50 vol % FA at 100 °C led to the formation of nanoparticles with approximately 30 nm in diameter. The origin of the nanoparticle formation is probably the suppression of the crystallization along the *c*-axis leading to a narrow particle size distribution (Figure 2 and Figure 3). Increasing the temperature to 120 °C resulted in the formation of longer nanoparticles (Figure S2, Supporting Information File 1). Unfortunately, all attempts to control the nanoparticle length failed and therefore we focused on the smaller nanoparticles. The best results were finally obtained in FA/DMF = 9:1 mixture at 100 °C for 96 h. The optimized synthesis was well reproducible and the size of the nanoparticles did not significantly differ from one batch to another.

**Figure 4:**
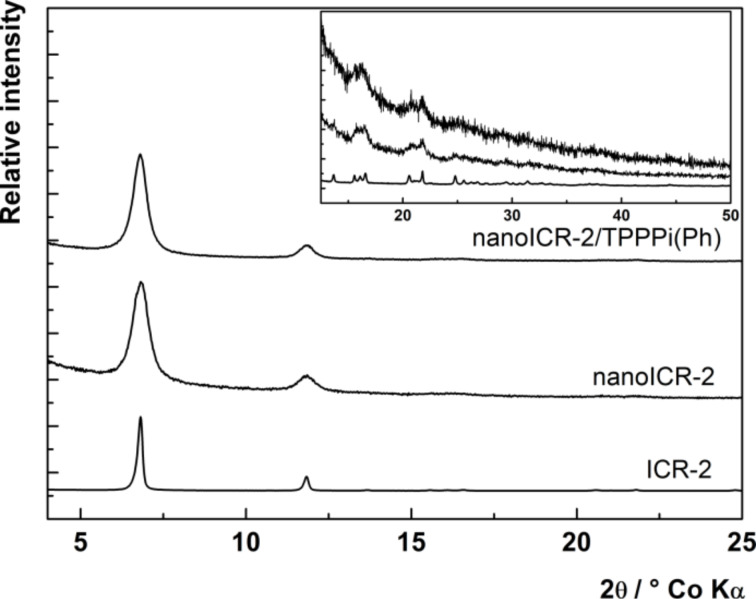
Powder XRD patterns of microcrystalline ICR-2 (bottom line), nanoICR-2 (middle line) and nanoICR-2/TPPPi(Ph) (top line), intensity of microcrystalline ICR-2 peaks were divided by 50.

To confirm the composition, nanoICR-2 was characterised by powder X-ray diffraction measurements (XRD), transmission electron microscopy (TEM), and dynamic light scattering (DLS). The powder XRD pattern depicted in Figure 4 clearly corresponds to the ICR-2 phase [25]. The size of the coherent diffraction domains of 29 nm was calculated from the broadening of the 110 and 020 diffractions using the Scherrer equation. The analysis of TEM images in Figure 2 (left) provides an average particle size of 28 nm, which is in good agreement with the results from the powder XRD analysis.

The dispersibility of nanoICR-2 in aqueous media and its surface charge potential were evaluated using dynamic light scattering (DLS, Figure S3A, Supporting Information File 1). In water, nanoICR-2 forms aggregates with a mean value of the size distribution of 87 ± 31 nm (by number, *Z*-average = 136 nm, PDI = 0.12). The zeta potential of nanoICR-2 in water is slightly positive with an average of 5 ± 5 mV (Figure S4A, Supporting Information File 1), evidencing that the prevailing terminal groups on the surface of nanoICR-2 are coordinatively unsaturated Fe cationic sites.

NanoICR-2 was modified with three anionic porphyrins: 5,10,15,20-tetrakis(4-methylphosphinatophenyl)porphyrin (TPPPi(Me)), 5,10,15,20-tetrakis(4-isopropylphosphinatophenyl)porphyrin (TPPPi(iPr)), and 5,10,15,20-tetrakis(4-phenylphosphinatophenyl)porphyrin (TPPPi(Ph)). We also tested commercially available 5,10,15,20-tetrakis(4-carboxyphenyl)porphyrin (TCPP). However, its binding was much weaker, which resulted in approximately 20-times lower porphyrin loading. This is probably due to weaker bond of the carboxylic group to Fe^3+^ in comparison with the phosphinic groups. The modification was done by shaking nanoICR-2 in DMF solution of the respective porphyrin. To achieve better biocompatibility, the modified nanoparticles were thoroughly washed with DMF and dispersed in absolute EtOH with a porphyrin concentration of 10^−4^ mol·L^−1^. To investigate the effects of porphyrin loadings, we prepared two additional samples where 50% and 25% of the original amount of TPPPi(Ph) was used for the modification (denoted nanoICR-2/TPPPi(Ph)½ and nanoICR-2/TPPPi(Ph)¼). This resulted in lower amounts of attached porphyrin to the nanoparticles, i.e., 6 × 10^−5^ and 3 × 10^−5^ mol·L^−1^ for nanoICR-2/TPPPi(Ph)½ and nanoICR-2/TPPPi(Ph)¼, respectively.

The ideal structure of ICR-2 does not contain free binding sites for anions and therefore the anionic porphyrins can bind only to the terminal Fe atoms located on the surface of the nanoparticles. In addition, the porphyrin units are larger (over 10 Å) than the pore diameter of ICR-2 (9 Å) and thus they cannot enter the pores. This hypothesis was confirmed by the fact that non-anionic 5,10,15,20-tetraphenylporphyrin does not bind to nanoICR-2. Also, when larger ICR-2 nanoparticles (prepared at 120 °C, Figure S2, Supporting Information File 1) were used the TPPPi(Ph) loading was an order of magnitude lower than for nanoICR-2.

The porphyrin-modified nanoICR-2 particles (nanoICR-2/porphyrin) were characterised by powder XRD, TEM, DLS, and UV–vis and fluorescence spectroscopy. The powder XRD patterns of all composites, depicted in Figure 4 and Figure S5 (Supporting Information File 1), do not show significant changes in comparison with that of the parent nanoICR-2. Also, the coherent diffraction domain of 30 nm is virtually unchanged from the parent nanoparticles. The analysis of TEM data confirmed the preservation of the particle size (29 nm on average), only the particle size distribution was broader (Figure 2).

DLS experiments with aqueous dispersions of nanoICR-2/porphyrin revealed the formation of nanoparticle aggregates, with mean values of the size distribution of 91 ± 23 nm (by number, *Z*-average = 198 nm, PDI = 0.24), 195 ± 90 nm (by number, *Z*-average = 291 nm, PDI = 0.26), and 128 ± 53 nm (by number, *Z*-average = 193 nm, PDI = 0.16) for TPPPi(Me), TPPPi(iPr), and TPPPi(Ph), respectively (Figure S3, Supporting Information File 1). These values are somewhat bigger than the size of the parent nanoICR-2 aggregates in water (87 ± 31 nm). Importantly, the zeta potential of the nanoICR-2/porphyrin aggregates switched to negative values: −20 ± 4 mV, −25 ± 5 mV, and −28 ± 5 mV for TPPPi(Me), TPPPi(iPr), and TPPPi(Ph), respectively (Figure S4, Supporting Information File 1). These results are consistent with the binding of the porphyrin phosphinate groups to the coordinatively unsaturated Fe cationic sites at the surface of the nanoparticles. Because of the nearly square planar geometry of the porphyrins it is probable that only 1–2 phosphinate groups are bonded to nanoICR-2 and therefore some of the phosphinate groups remains unbound and induce the negative zeta potentials. In agreement with this assumption, the zeta potentials decrease with decreasing porphyrin loading to −22 ± 3 mV and −17 ± 4 mV for nanoICR-2/TPPPi(Ph)½ and nanoICR-2/TPPPi(Ph)¼, respectively.

We also tested the stability of nanoICR-2/porphyrin in PBS media. Even though ICR-2 can stand PBS treatment in its microcrystalline form, when nanoparticles of either nanoICR-2 or nanoICR-2/porphyrin were treated for 4 h in PBS it resulted in amorphisation of the ICR-2 nanoparticles, and in the case of nanoICR-2/porphyrin in partial dissolution of the porphyrin.

**Figure 5:**
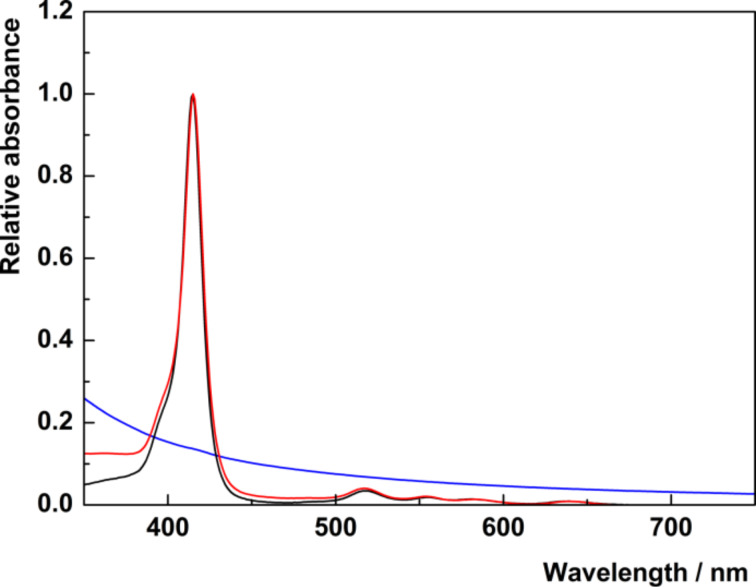
Normalized UV–vis spectra of nanoICR-2 (blue), TPPPi(Ph) (black), and nanoICR-2/TPPPi(Ph) (red) in EtOH solution.

In order to ascertain the effects of the MOF structure on the porphyrin units, UV–vis absorption and fluorescence spectra were measured and compared with the corresponding spectra of the free porphyrins. The absorption spectra show characteristic absorption bands of metal-free porphyrins: the Soret band at 415 nm and four Q-bands in the region between 500 and 650 nm. The comparison of the absorption spectra of free TPPPi(Ph) and corresponding nanoICR-2/TPPPi(Ph) nanoparticles (Figure 5) demonstrates that the position and shape of the absorption bands do not change after binding of the porphyrin units onto the nanoparticles. The same observation is valid for both nanoICR-2/TPPPi(Ph)½ and nanoICR-2/TPPPi(Ph)¼ (Figure S6, Supporting Information File 1), indicating that the formation of porphyrin aggregates on the surface of the ICR-2 nanoparticles is not controlled by the amount of bound porphyrins. On the other hand, the Soret bands of nanoICR-2/TPPPi(Me) and nanoICR-2/TPPPi(iPr) (Figure S7 and Figure S8, Supporting Information File 1) are broadened and red shifted because of partial porphyrin aggregation. The magnitude of aggregation is in the order TPPPi(Ph) < TPPPi(iPr) ≈ TPPPi(Me).

When excited at 415 nm, the dispersions of nanoICR-2/porphyrin in EtOH exhibit red fluorescence with two bands at 655 and 720 nm (Figure S9, Supporting Information File 1). The fluorescence quantum yields of approximately 0.01 are rather low, when compared with free porphyrins in the same solvent (Φ_f_ = 0.07). The lower Φ_f_ values can be attributed to non-radiative quenching of the excited singlet states due to the partial aggregation of the porphyrins at the surface of nanoICR-2 and the proximity of iron atoms constituting the ICR-2 structure. It is worth noting that the porphyrin loading does not affect the fluorescence quantum yields.

### Photobiological properties

#### Cellular uptake and intracellular localization

**Figure 6:**
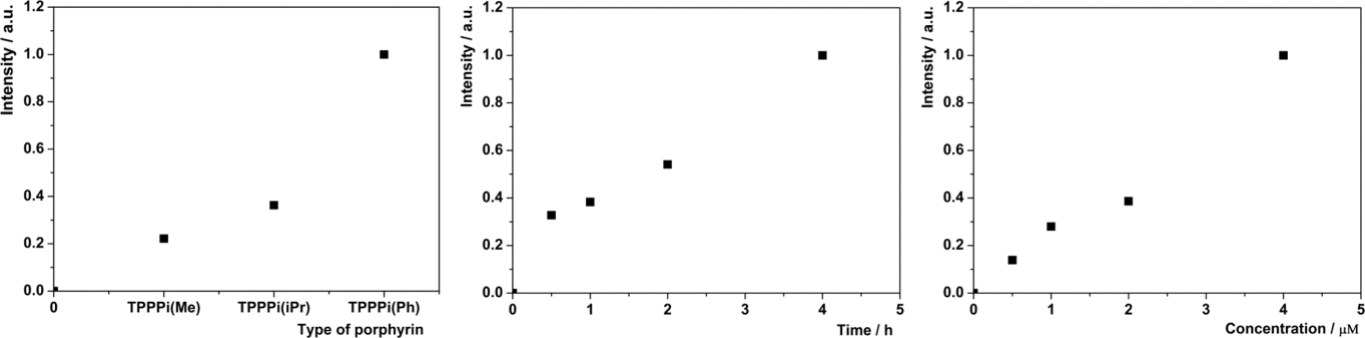
(A) Comparison of the cellular uptake of different types of nanoICR-2 with porphyrin concentration of 1 μM; (B) time dependence of the cellular uptake of nanoICR-2/TPPPi(Ph) with porphyrin concentration of 1 μM; (C) concentration dependence of the cellular uptake of nanoICR-2/TPPPi(Ph).

**Figure 7:**
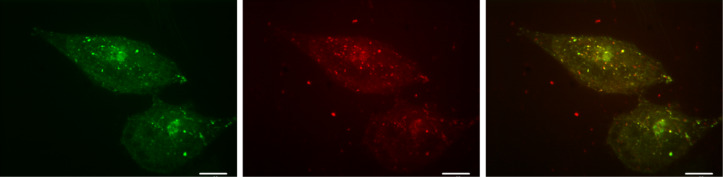
Confocal microscopy of HeLa cells incubated with 2 µM nanoICR-2/TPPPi(Ph) for 24 h: LysoTracker Green (left); nanoICR-2/TPPPi(Ph) (middle); overlay (right). The white scale bars correspond to 10 µm.

HeLa cells were treated with the nanoparticles in Eagle's Minimum Essential Medium (EMEM) without foetal bovine serum to avoid modification of the particle surface properties by nonspecific binding of serum albumin. The cellular uptake of the nanoparticles was quantified by flow-cytometry analysis of porphyrin fluorescence associated with the cells. Figure 6A shows the rate of internalization of the photosensitizers into the cells with different modifications of nanoICR-2. The highest cellular uptake was observed for nanoICR-2/TPPPi(Ph), followed by nanoICR-2/TPPPi(iPr) and nanoICR-2/TPPPi(Me). The most efficiently accumulating sample nanoICR-2/TPPPi(Ph) was therefore selected for determination of the uptake kinetic. As shown in Figure 6B, the incubation of the cells with a fixed amount of the nanoparticles for different time periods revealed significant uptake already after 30 min and the concentration increased gradually up to 4 h. For this reason, further photobiological experiments were performed after 4 h of incubation with the nanoparticles. The cellular uptake upon incubation with different concentrations of the nanoparticles yielded almost linear dose dependence (Figure 6C). Furthermore, the intracellular localization of nanoICR-2/TPPPi(Ph) was investigated using confocal microscopy. Figure 7 clearly shows that the nanoparticles accumulate in intracellular vesicles, which strongly co-localize with the fluorescent marker of lysosomes. This is similar to the results of a previous study performed with PCN-222 nanoparticles [22].

#### Toxicity and phototoxicity studies

**Figure 8:**
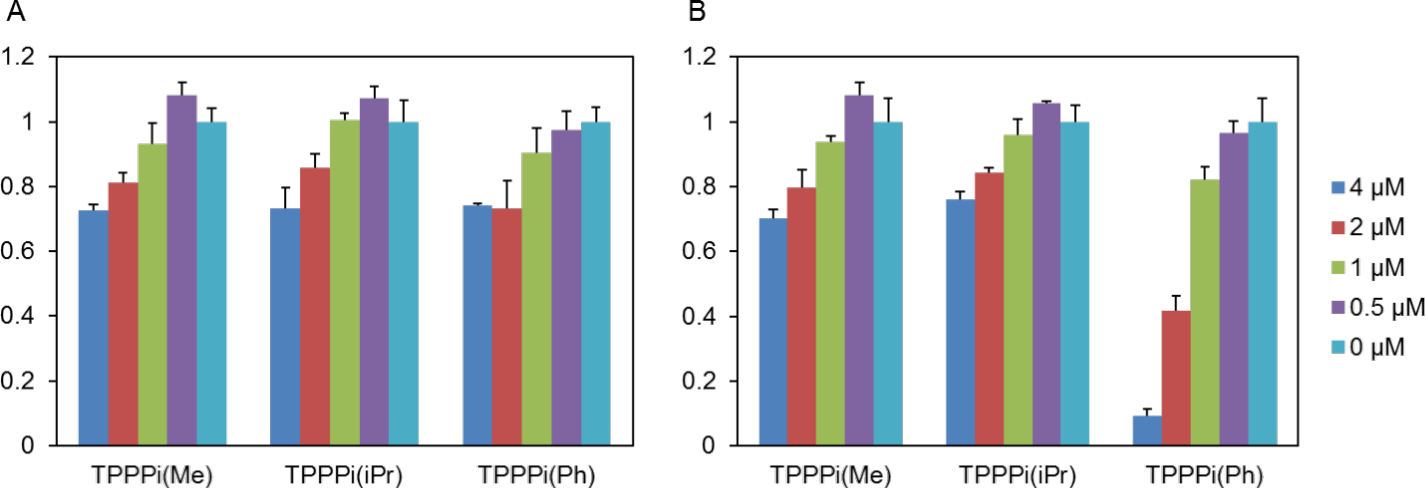
Relative viability of HeLa cells incubated for 4 h with specified concentrations of nanoICR-2/porphyrin in the dark (A) or irradiated with a halogen lamp for 15 min (B). Note: The results labelled 0 µM belong to the control experiments in which cells were irradiated in the absence of nanoICR-2/porphyrin.

Dark toxicity of the porphyrin-modified nanoICR-2 was investigated on HeLa cells in EMEM without serum in the presence of 0.5–4 µM nanoparticles (with respect to porphyrin) for 4 h followed by incubation in full culture medium without phenol red for 24 h. At concentrations used for the experiments, only limited suppression of cellular metabolic activity was observed for all three porphyrins (Figure 8A). On the other hand, when the cells were irradiated with a water-filtered halogen light, only nanoICR-2/TPPPi(Ph) exhibited a clear phototoxic effect. The IC_50_ value of this sample was 1.8 ± 0.5 μM. Interestingly, nanoICR-2/TPPPi(iPr) and nanoICR-2/TPPPi(Me) did not reveal any phototoxic effect (Figure 8B). The results of phototoxicity tests well correspond with the cellular uptake values, which were the highest for nanoICR-2/TPPPi(Ph). A comparison of PDT activity with other systems can be made only under identical conditions (e.g., irradiation wavelength, time, dose). We can compare the activity of nanoICR-2/TPPPi(Ph) with the activity of previously studied PCN-222 nanoparticles where both systems display comparable activity [22].

## Conclusion

In the context of photodynamic therapy, we present composite materials based on nanoparticles of the ICR-2 metal-organic framework decorated with phosphinic acid-substituted porphyrins. These substituted porphyrins showed superior affinity towards the Fe-MOF ICR-2 in comparison with the well-known tetracarboxyphenyl porphyrin, and this feature allows for superior photosensitizer loading of the nanoparticles. The porphyrins retain part of their photophysical properties including production of singlet oxygen. Interestingly, the photodynamic activity on HeLa cells strongly depends on the R substituent at the P atom, i.e., only the phenyl substituent (TPPPi(Ph)) ensured high phototoxicity, comparable with the best MOF systems. This feature does not seem to arise from the differences in singlet oxygen photosensitizing ability [26], but can be assigned to the differences in the biological properties provided by the substituent of the unbounded phosphinic groups at the surface of the composite nanoparticles. Unfortunately, the elucidation of the structure–activity relationship is rendered difficult due to the instability of the nanoICR-2/porphyrin nanoparticles in PBS media, as also observed for Zr-based MOFs.

## Experimental

**Materials:** *N*,*N-*dimethylformamide (DMF, Lach-Ner, Czech Republic), absolute ethanol (EtOH, Fischer Sci.), FeCl_3_·6H_2_O, formamide, and phosphate-buffered saline suitable for cell culture (all Sigma-Aldrich) were used as purchased. Phenylene-1,4-bis(methylphosphinic acid) was prepared according to [25]. Phosphinic acid porphyrins 5,10,15,20-tetrakis(4-methylphosphinatophenyl)porphyrin (TPPPi(Me)), 5,10,15,20-tetrakis(4-isopropylphosphinatophenyl)porphyrin (TPPPi(iPr)), and 5,10,15,20-tetrakis(4-phenylphosphinatophenyl)porphyrin (TPPPi(Ph)) were prepared according to [26].

**Synthesis of nanoICR-2:** Into a 20mL vial (Wheaton) was added 5.4 mg (0.02 mmol) FeCl_3_·6H_2_O, 9.4 mg (0.04 mmol) PBPA, 1 mL DMF, and 9 mL formamide. The vial was tightly closed and immersed into a programmable oven (Memmert UF30 Plus) for 96 h at 100 °C (heat ramp 1 h and cooling down 6 h). The resulting mixture was centrifuged (Hettich Rotina 380R, 11000 rpm for 5 min) and washed three times with distilled water and two times with absolute EtOH; dispersion of ICR-2 nanoparticles in EtOH (approximately 5 mL) was obtained.

**Modification of nanoICR-2 with porphyrins:** The whole batch of nanoICR-2 dispersion in EtOH prepared above was centrifuged again (11000 rpm, 5 min), the solvent was decanted, and nanoICR-2 was dispersed in 5 mL of DMF. Separately, 2.5 mmol of each porphyrin was dissolved in 10 mL of DMF, the solution was added to the nanoICR-2 dispersion, and the mixture was shaken at RT overnight (16 h). Then, the mixture was centrifuged (11000 rpm, 5 min.) and washed three times with absolute EtOH to remove the excess of porphyrin. Finally, nanoICR-2/TPPPi(Me), nanoICR-2/ TPPPi(iPr), and nanoICR-2/TPPPi(Ph) were dispersed and stored in absolute EtOH (ca. 10 mL) at a concentration of 10^−4^ mol·L^−1^. The samples with lower porphyrin loading were prepared analogously, only the amount of the TPPPi(Ph) in the DMF solution was 1.25 mmol (nanoICR-2/TPPPi(Ph)½) and 0.625 mmol (nanoICR-2/TPPPi(Ph)¼). This led to lower porphyrin concentrations in the resulting colloids of 6 × 10^−5^ mol·L^−1^ and 3 × 10^−5^ mol·L^−1^ for nanoICR-2/TPPPi(Ph)½ and nanoICR-2/ TPPPi(Ph)¼, respectively.

**Stability studies:** NanoICR-2/TPPPi(Ph) in EtOH were centrifuged (11000 rpm, 5 min), redispersed in 10 mL of PBS, and shaken for 4 h at RT. The resulting mixture was centrifuged (11000 rpm, 5 min), washed three times with absolute EtOH, and air-dried before powder XRD measurement.

**Instrumental methods:** Powder X-ray diffraction (XRD) was measured using a PANalytical X'Pert PRO diffractometer in the reflexion setup equipped with a conventional Co X-ray tube (40 kV, 30 mA). Qualitative analysis was performed with the HighScorePlus software package (PANalytical, Almelo, The Netherlands, version 3.0) and the JCPDS PDF-2 database [27]. UV–vis absorption spectra of the dispersions were recorded on a Perkin Elmer Lambda 35 spectrometer. High-resolution transmission electron microscopy (TEM) was carried out on a JEOL JEM 3010 microscope operated at 300 kV (LaB_6_ cathode, point resolution 1.7 Å) with an Oxford Instruments Energy Dispersive X-ray (EDX) detector. The particle size distributions and zeta potentials in water were determined by dynamic light scattering (DLS) using a particle size analyser Zetasizer Nano ZS (Malvern, UK). Fluorescence spectra and absolute fluorescence quantum yields, Φ_L_, were measured using a Quantaurus QY C11347-1 spectrometer (Hamamatsu, Japan).

**Cultivation of the cells:** The human cervix carcinoma HeLa cell line was cultivated in the Eagle's Minimum Essential Medium (EMEM; Sigma-Aldrich) supplemented with 0.5 mM glutamine and 5% foetal bovine serum (full culture medium) at 37 °C in atmosphere containing 5% CO_2_.

**Phototoxicity and dark toxicity studies:** The cells were seeded onto 96-well plates in full culture medium. Next day, the cells were exposed to 0.5–4 μM equivalent of the nanoparticles in the fresh medium without foetal bovine serum for 4 h. A final concentration of EtOH in the culture medium was less than 4 % v/v. After incubation, the medium was changed for full culture medium without phenol red and the cells were immediately irradiated by a 150 W halogen lamp (Thorlabs) with a water filter for 15 min (45 mW·cm^−2^). After another 24 h, a viability of the cells was assayed by the resazurin assay (Sigma-Aldrich). Dark toxicity experiments were performed in the same way in the dark.

**Confocal microscopy:** HeLa cells were seeded onto dishes with a glass bottom (MatTek) in full culture medium. After 24 h, the cells received fresh medium without serum and phenol red, and were mixed with the nanoparticles of 2 μM total concentration. After 4 h, the cells were washed and stained with LysoTracker Green (Thermo Fisher Scientific) and inspected with a spinning disc confocal microscope (Revolution XD, Andor). The excitation wavelengths used for monitoring of nanoparticles and lysosomes were 405 nm and 488 nm, respectively. During the confocal microscopy, the cells were maintained at 37 °C and 5% CO_2_ atmosphere.

**Flow cytometry:** The cells were plated onto 6-well plates in full culture medium. The next day, they were treated with indicated amount of nanoparticles for the indicated period of time in the fresh medium without serum. Then, the plates were washed with PBS and trypsinized. Uptake of MOFs was measured by flow cytometry analysis with excitation and emission recorded at 405 nm and 655–685 nm, respectively (BD FACSaria III).

## Supporting Information

File 1 Additional experimental data.
