# Supplementary material for: The nanoscaled metal-organic framework ICR-2 as a carrier of porphyrins for photodynamic therapy
Source: Beilstein J Nanotechnol. 2018 Nov 30;9:2960–7. doi: 10.3762/bjnano.9.275 (PMC6278752; doi:10.3762/bjnano.9.275)
Supplement: File 1 — Additional experimental data. [file Beilstein_J_Nanotechnol-09-2960-s001.pdf]

BEILSTEIN JOURNAL OF NANOTECHNOLOGY

Supporting Information

for

The nanoscaled metal-organic framework ICR-2 as a carrier of porphyrins for photodynamic therapy

Jan Hynek, Sebastian Jurík, Martina Koncošová, Jaroslav Zelenka, Ivana Křížová, Tomáš Ruml, Kaplan Kirakci, Ivo Jakubec, František Kovanda, Kamil Lang and Jan Demel

Beilstein J. Nanotechnol. **2018,** *9,* 2960–2967. doi:10.3762/bjnano.9.275

Additional experimental data

License and Terms: This is a supporting information file under the terms of the Creative Commons Attribution License (http://creativecommons.org/licenses/by/4.0). Please note that the reuse, redistribution and reproduction in particular requires that the authors and source are credited.
The license is subject to the *Beilstein Journal of Nanotechnology* terms and conditions: (https://www.beilstein-journals.org/bjnano)

Contents

Figure S1: Powder XRD patterns of ICR-2 and UiO-66 treated in PBS for 4 h.

Figure S2: TEM image of nanoparticles prepared at 120°C.

Figure S3: Particle size distribution by number of aqueous dispersions of nanoICR-2 and nanoICR-2/porphyrin samples.

Figure S4: Zeta potential distribution of water dispersions of nanoICR-2 and nanoICR-2/porphyrin samples.

Figure S5: Powder XRD patterns of parent nanoICR-2 and nanoICR-2/porphyrin samples.

Figure S6: UV–vis spectra of free TPPPi(Ph), nanoICR-2/TPPPi(Ph), nanoICR-2/TPPPi(Ph)½ and nanoICR-2/TPPPi(Ph)¼.

Figure S7: UV–vis spectra of free TPPPi(Me) and nanoICR-2/TPPPi(Me).

Figure S8: UV–vis spectra of free TPPPi(iPr) and nanoICR-2/TPPPi(iPr).

Figure S9: Normalized luminescence spectra of nanoICR-2/TPPPi(Me), nanoICR-2/TPPPi(iPr), and nanoICR-2/TPPPi(Ph) in EtOH solution.

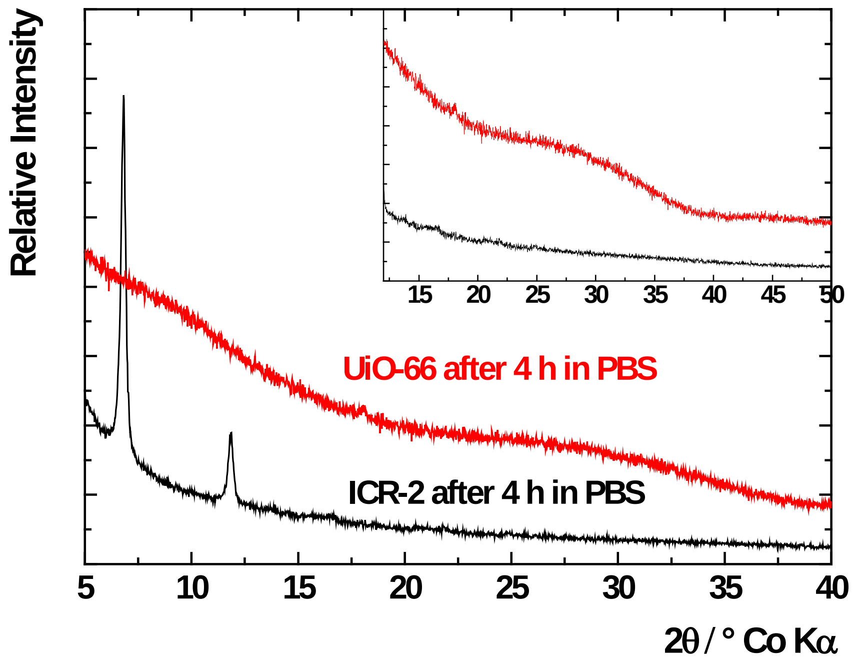

Figure S1: Powder XRD patterns of ICR-2 (black) and UiO-66 (red) both treated in PBS for 4 h.

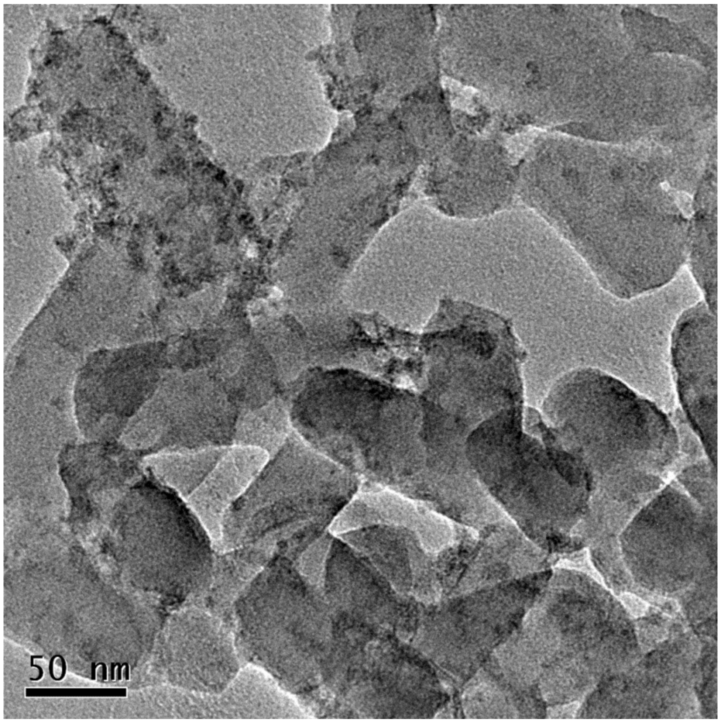

Figure S2: TEM image of nanoparticles prepared at 120 °C.

A

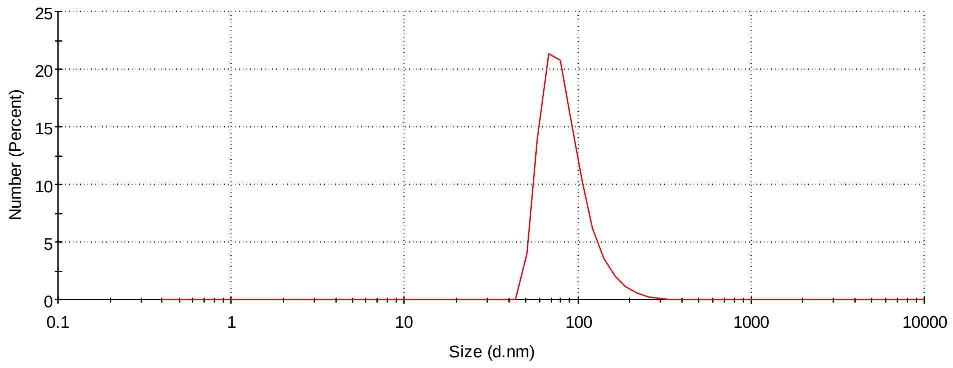

Number (Percent)
25
20
15
10
5
0
0.1
1
10
100
1000
10000
Size (d.nm)

B

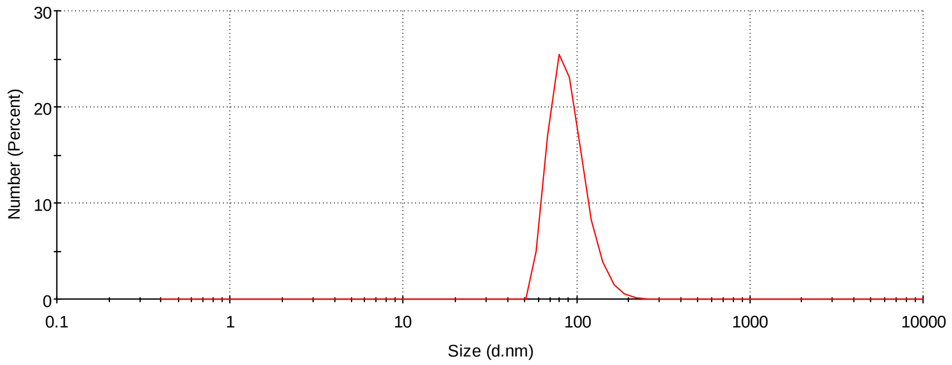

Number (Percent)
30
20
10
0
0.1
1
10
100
1000
10000
Size (d.nm)

C

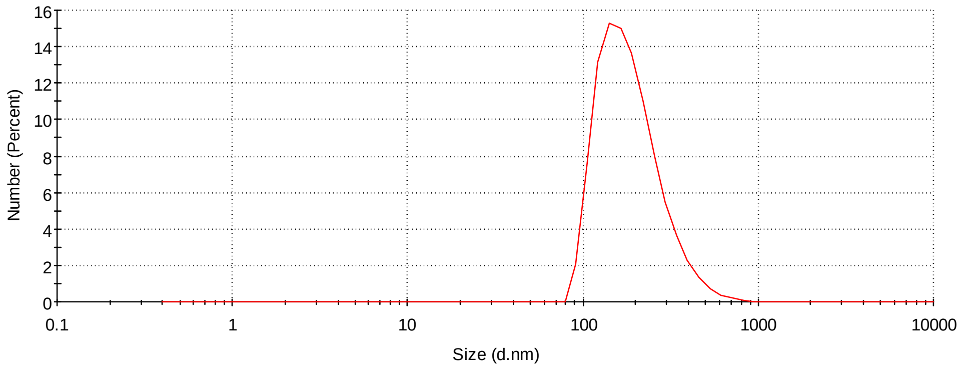

Number (Percent)
16
14
12
10
8
6
4
2
0
0.1
1
10
100
1000
10000
Size (d.nm)

D

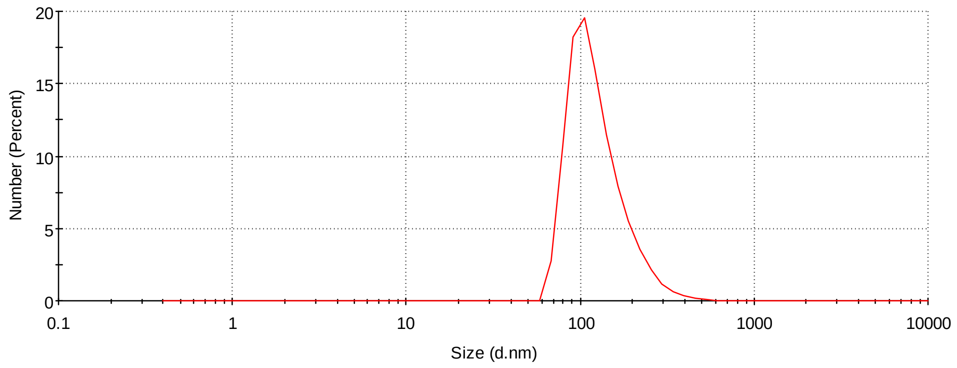

E

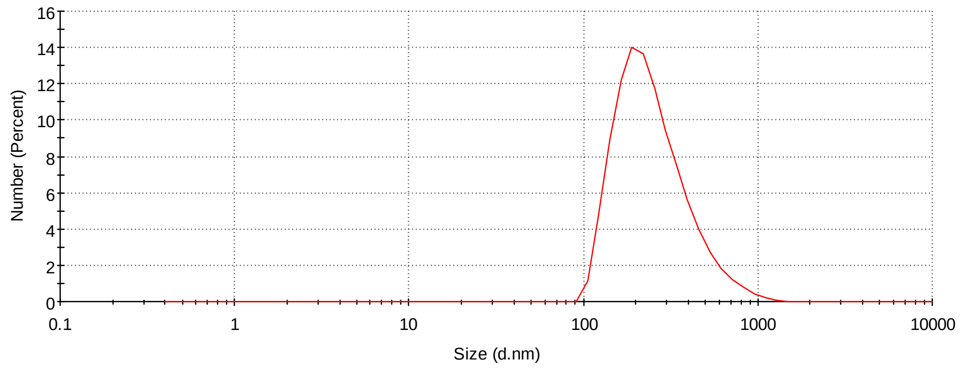

F

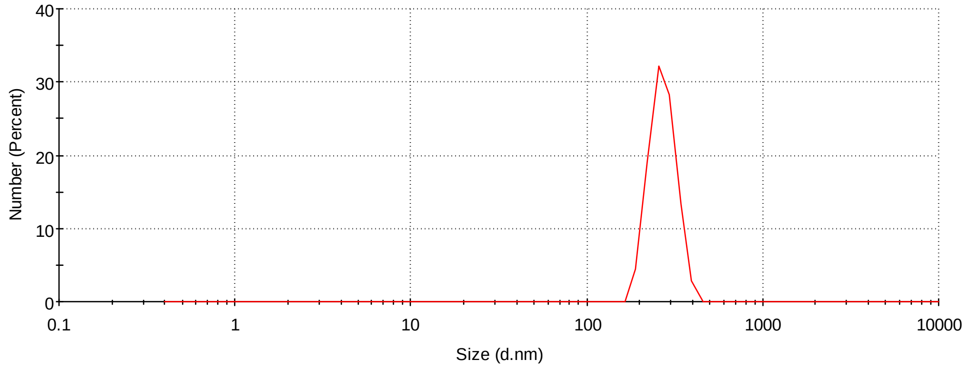

Figure S3: Particle size distribution by number of aqueous dispersions of nanoICR-2 (A), nanoICR-2/TPPPi(Me) (B), nanoICR-2/TPPPi(iPr) (C), nanoICR-2/TPPPi(Ph) (D), nanoICR-2/TPPPi(Ph)½ (E), and nanoICR-2/TPPPi(Ph)¼ (F).

A

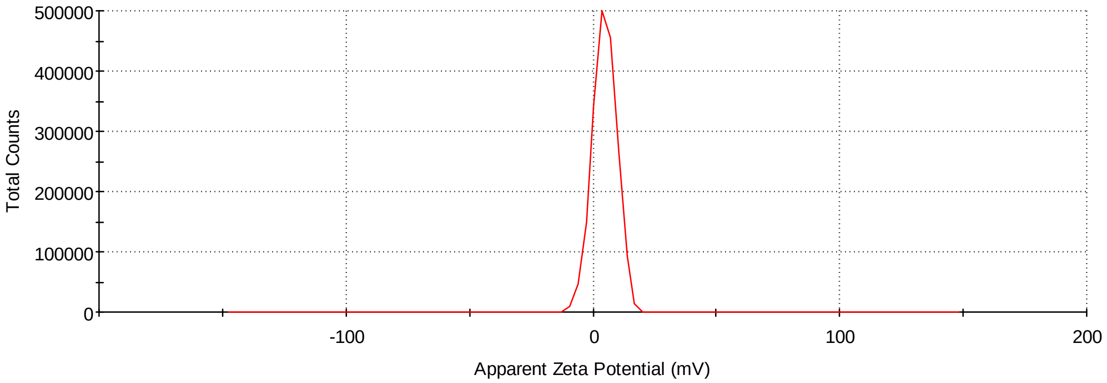

500000
400000
300000
200000
100000
0
Total Counts
-100
0
100
200
Apparent Zeta Potential (mV)

B

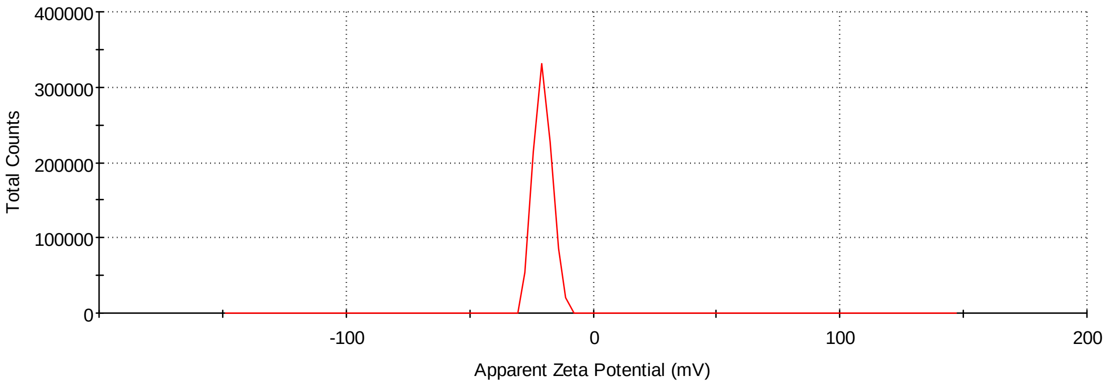

400000
300000
200000
100000
0
Total Counts
-100
0
100
200
Apparent Zeta Potential (mV)

C

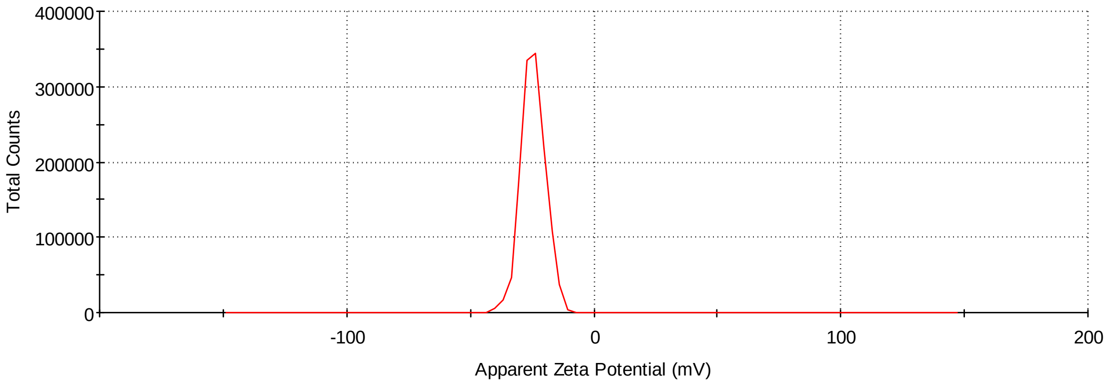

400000
300000
200000
100000
0
Total Counts
-100
0
100
200
Apparent Zeta Potential (mV)

D

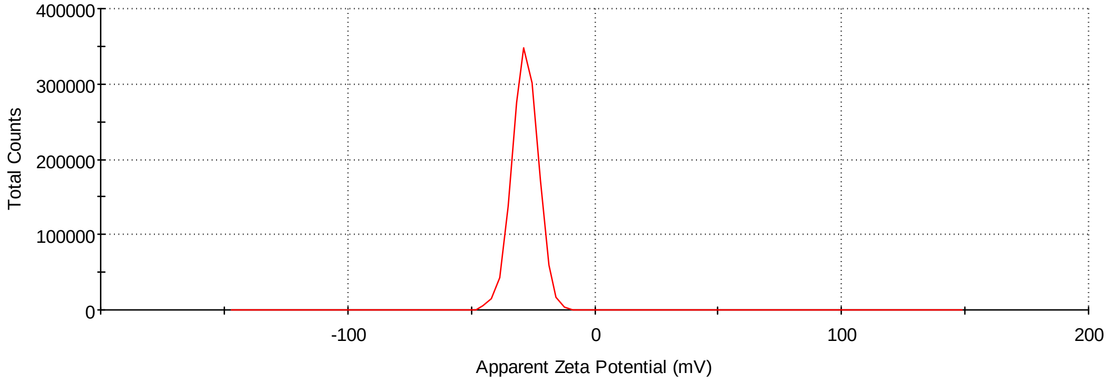

E

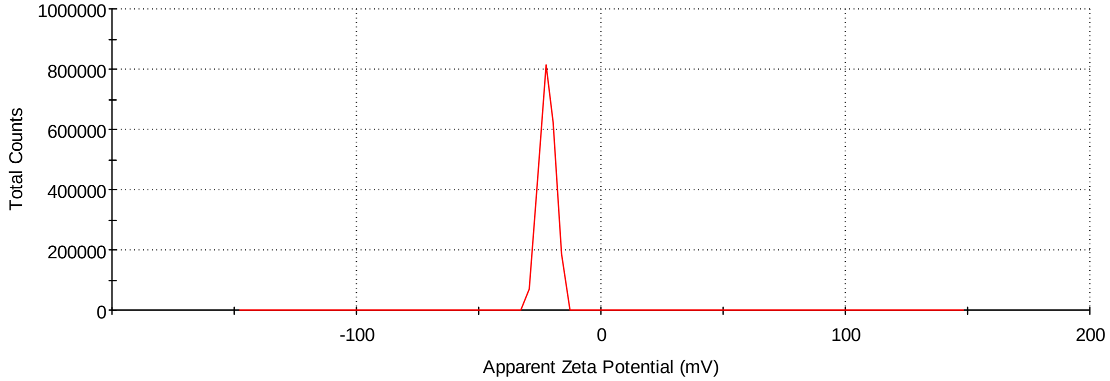

F

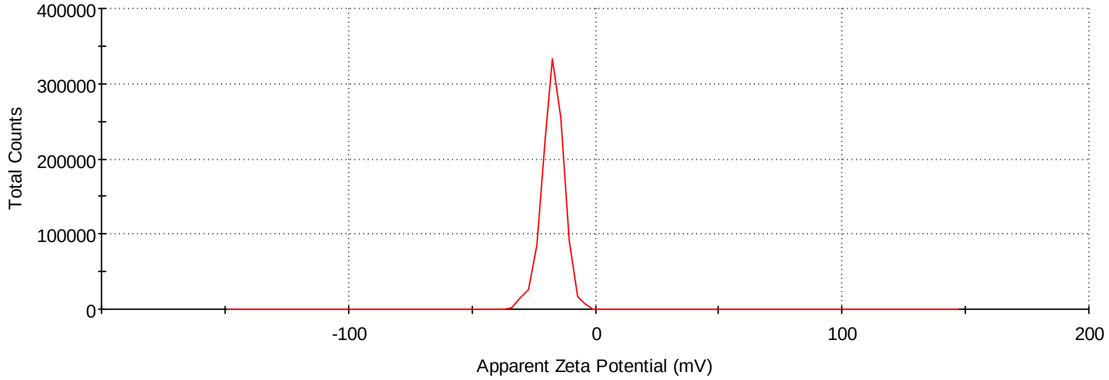

Figure S4: Zeta potential distribution of water dispersions of nanoICR-2 (A), nanoICR-2/TPPPi(Me) (B), nanoICR-2/TPPPi(iPr) (C), nanoICR-2/TPPPi(Ph) (D), nanoICR-2/TPPPi(Ph)½ (E), and nanoICR-2/TPPPi(Ph)¼ (F).

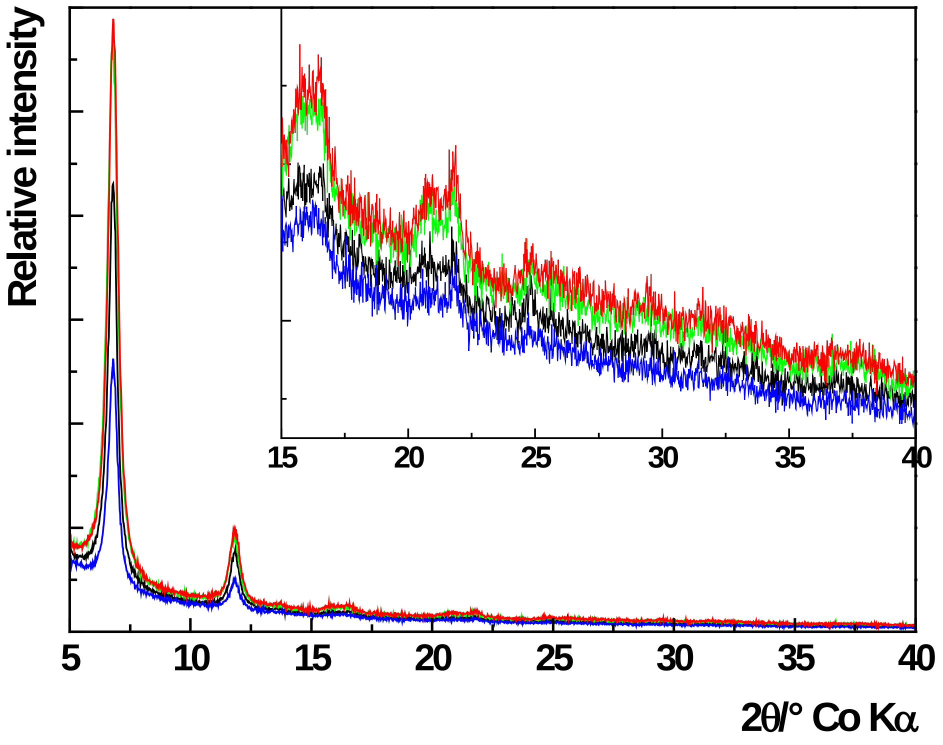

Figure S5: Powder XRD patterns of parent nanoICR-2 (black), nanoICR-2/TPPPi(Me) (green), nanoICR-2/TPPPi(iPr) (blue) and nanoICR-2/TPPPi(Ph) (red).

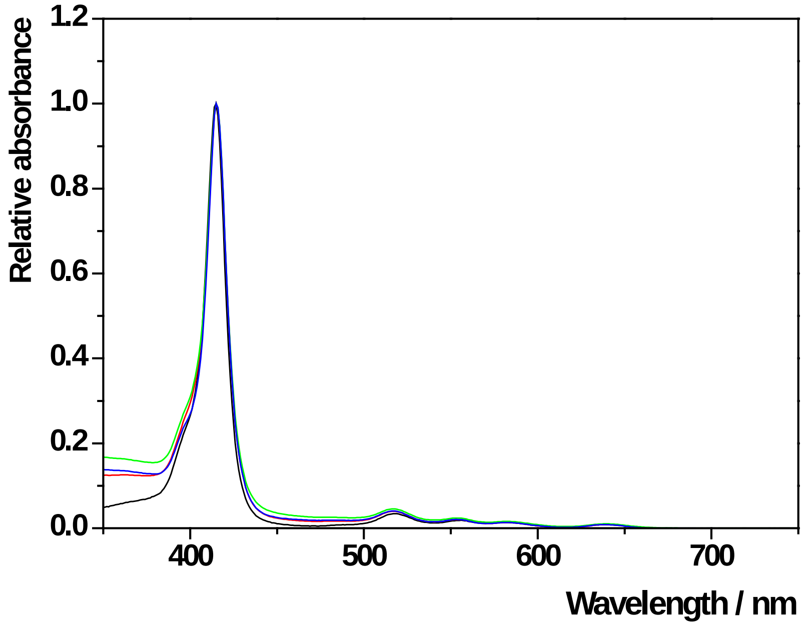

Figure S6: UV–vis spectra of free TPPPi(Ph) (black), nanoICR-2/TPPPi(Ph) (red), nanoICR-2/TPPPi(Ph)½ (blue) and nanoICR-2/TPPPi(Ph)¼ (green) in EtOH.

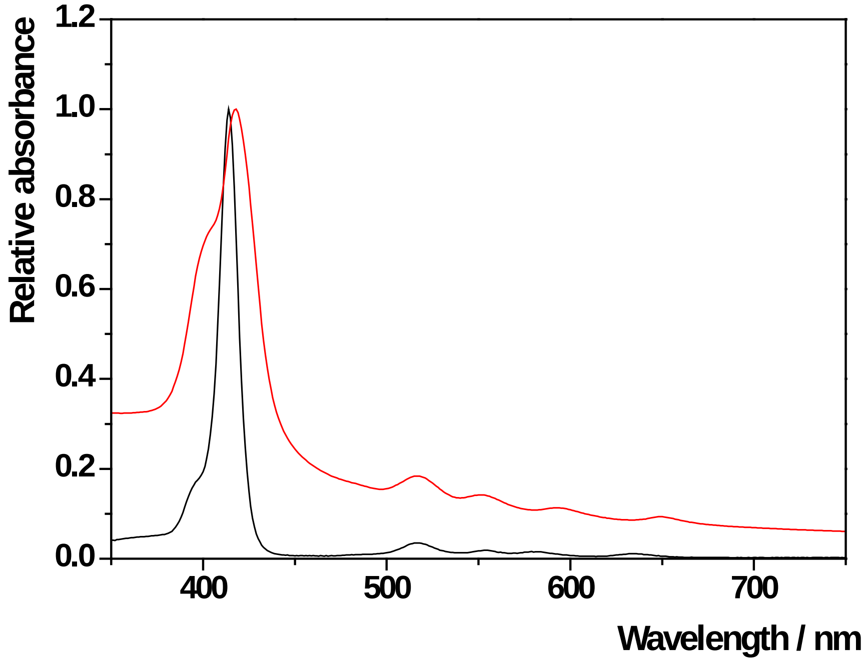

Figure S7: UV–vis spectra of free TPPPi(Me) (black) and nanoICR-2/TPPPi(Me) (red) in EtOH.

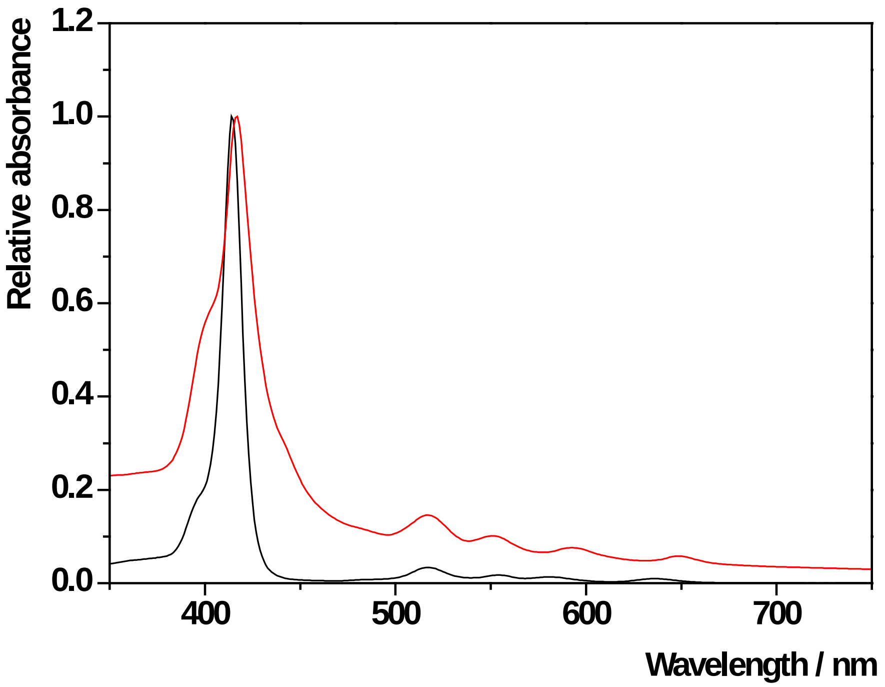

Figure S8: UV–vis spectra of free TPPPi(iPr) (black) and nanoICR-2/TPPPi(iPr) (red) in EtOH.

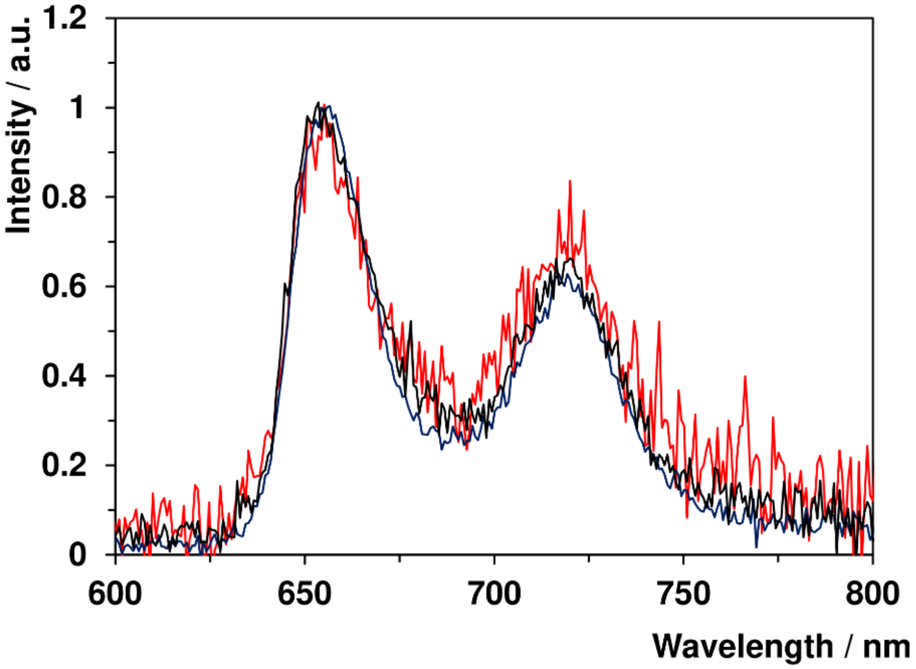

Figure S9: Normalized fluorescence spectra of nanoICR-2/TPPPi(Me) (red), nanoICR-2/TPPPi(iPr) (blue), and nanoICR-2/TPPPi(Ph) (black) in EtOH solution.
